# Comparison of anterior and posterior approaches in Treating odontoid fractures: a meta-analysis and systematic review

**DOI:** 10.3389/fsurg.2023.1125665

**Published:** 2023-06-12

**Authors:** Xianguo Bao, Yingjun Chen, Chen Guo, Shuai Xu

**Affiliations:** ^1^ Spinal Surgery, Nanjing Lishui People’s Hospital, Nanjing, China; ^2^ Spinal Surgery, Peking University People’s Hospital, Beijing, China

**Keywords:** odontoid fracture, anterior approach, posterior approach, fusion rate, complications, meta-analysis

## Abstract

**Background:**

Odontoid fractures account for 15%–20% of cervical injuries. Although the operation methods vary in different types, the superiority of overall outcomes of the anterior approach (AA) and posterior approach (PA) in treating odontoid fractures still remains controversial. Thus, a meta-analysis was performed comparing AA and PA for these fractures.

**Methods:**

The relevant studies were searched in PubMed/MEDLINE, Cochrane Library, EMBASE, China Biological Medicine (CBM), and Wanfang Database from the onset of conception to June 2022. Prospective or retrospective comparative studies on AA and PA for odontoid fractures were screened, referring to fusion rates (primary outcomes), complications, and postoperative mortality rates. A meta-analysis of the primary outcomes and a systematic review of other outcomes were performed; the procedure was conducted with Review Manager 5.3.

**Results:**

Twelve articles comrising 452 patients were included, and all publications were retrospective cohort studies. The average postoperative fusion rate was 77.5 ± 17.9% and 91.4 ± 13.5% in AA and PA, respectively, with statistical significance [OR = 0.42 (0.22, 0.80), *P* = 0.009]. Subgroup analysis showed a difference in fusion rates between AA and PA in the elderly group [OR = 0.16 (0.05, 0.49), *P* = 0.001]. Five articles referred to postoperative mortality, and the mortality rates of AA (5.0%) and PA (2.3%) showed no statistical difference (*P* = 0.148). Nine studies referred to complications, with a rate of 9.7%. The incidence of complications in AA and PA groups was comparable (*P* = 0.338), and the incidence of nonfusion and complications was irrelevant. The prevalent cause of death was myocardial infarction. The time and segmental movement retention of AA were possibly superior to those of PA.

**Conclusion:**

AA may be superior in regard to operation time and motion retention. There was no difference in complications and mortality rates between the two approaches. The posterior approach would be preferred in consideration of the fusion rate.

## 1. Introduction

An odontoid fracture is the most common acute injury of the axis, accounting for 15%–20% of the cervical spine injury and showing an upward tendency in nearly 20 years (1). Odontoid fractures were categorized into three types by Anderson and D'Alonzo in 1974, among which type II fractures were the majority, with a proportion from 65% to 74% (2).

According to the point by Yoganandan and Osti, the main causes of odontoid fractures were traumatic injury and osteoporosis (3, 4), where type II and type III fractures were commonly treated with surgeries due to poor blood supplementation. Generally, surgical indications include fracture displacements longer than 5 mm, angulation deformities larger than 10°, and the combination with neurological dysfunction. Shilpakar et al. believed that this kind of injury is more inclined to receive operation (5) and the symoptoms might be relaxed unless patients are fit for general anesthesia, or else the conservative treatment would lead to higher potential mortality. Surgical approaches are usually divided into an anterior approach (AA) and a posterior approach (PA). However, based on perspective advantages, the current evidence on the selection of AA and PA remains controversial. Experts who supported AA suggested that it was directly exposed to the fracture site for fixation and retained the motion of the C1–2 unit (6), while others confirmed that PA could be applied for various fracture types, with wider application and better stability (7). Based on the superiority defects reported on both procedures, the overall efficacy was still undetermined.

It was believed that the fusion rate, to a great extent, reflects the stability and potential risk of spinal cord injury. Some publications reported a nonfusion rate ranging from 0% to 27% (8), and the fusion rate of PA was higher than that of anterior selection (7), but the constriction of the number and quality of literature weakened the conviction. For decades, there have been publicized systematic reviews on comparing the fusion rates of odontoid fractures (9), but a few literature works account for the overall efficacy by pooling data from both procedures. Therefore, based on previous studies, we aimed to perform a meta-analysis and systematic review to identify the overall efficacy of AA and PA.

## 2. Materials and methods

The studies were mainly retrospective therapeutic studies. Patients with odontoid fractures, mainly diagnosed as type II and type III fractures with surgical indications, were reviewed, and the intervention was AA and PA for odontoid fractures.

The studies were searched in PubMed/MEDLINE, Cochrane Library, EMBASE, China Biological Medicine (CBM), and Wanfang Database from January 1988 to June 2022. The keywords were odontoid fracture OR odontoid process fracture OR dens fracture AND odontoid screw OR anterior screw OR dens osteosynthesis AND C1–C2 fusion OR transarticular OR wiring OR posterior cervical arthrodesis OR atlantoaxial arthrodesis.

The inclusion criteria were the following: (1) the article was a prospective or retrospective therapeutic study; (2) the article was a comparative study referring to AA and PA; (3) the outcomes were about postoperative fusion rates or other parameters such as the incidence of complications, postoperative mortality rate, and so on; and (4) the number of samples in each group was at least 3. The exclusion criteria were the following: (1) the studies were case reports, reviews, or meta-analyses; (2) the literature merely referred to odontoid fractures with no surgery; and (3) the comparisons were not performed between AA and PA.

Two reviewers screened and evaluated the literature per the inclusion criteria, and the discrepancy was uniformed by a third person. Two individuals extracted data independently. Data extraction included the outcomes mainly containing surgery time, fusion rates, intra- and postoperative complications, mortality, and cervical activity.

Two reviewers independently evaluated the quality with the Newcastle–Ottawa quality assessment scale (NOS), which was used on nonrandomized controlled literature in meta-analysis. NOS refers to the literature selection, comparability, and outcome with a total score of nine. The 0−3, 4–6, and 7–9 scores represent low quality, middle quality, and high quality, respectively, where the articles with scores above 5 could be included in the study.

The meta-analysis was performed to assess the postoperative fusion rate, and the subgroup analysis was performed to assess the postoperative fusion rate in terms of age. The heterogeneity was measured by *I*^2^, where a value of *I*^2^ lower than 50% suggested a small heterogeneity, which could be addressed with fixed-effects models. The results of dichotomous variables were shown as odds ratios (ORs) and 95% confidence intervals (CIs) by the Mantel–Haenszel method, and *P*-values <0.05 showed a statistical difference. The meta-analysis was performed using Review Manager software, version 5.3 (The Cochrane Collaboration, Oxford, UK). The *χ*^2^ test was used for comparing mortality and complication rates between groups, which was analyzed by IBM SPSS Statistics 22.0 (International Business Machines Corporation, Armonk, NY, USA).

## 3. Results

### 3.1. Characteristics of included studies

**Figure 1:**
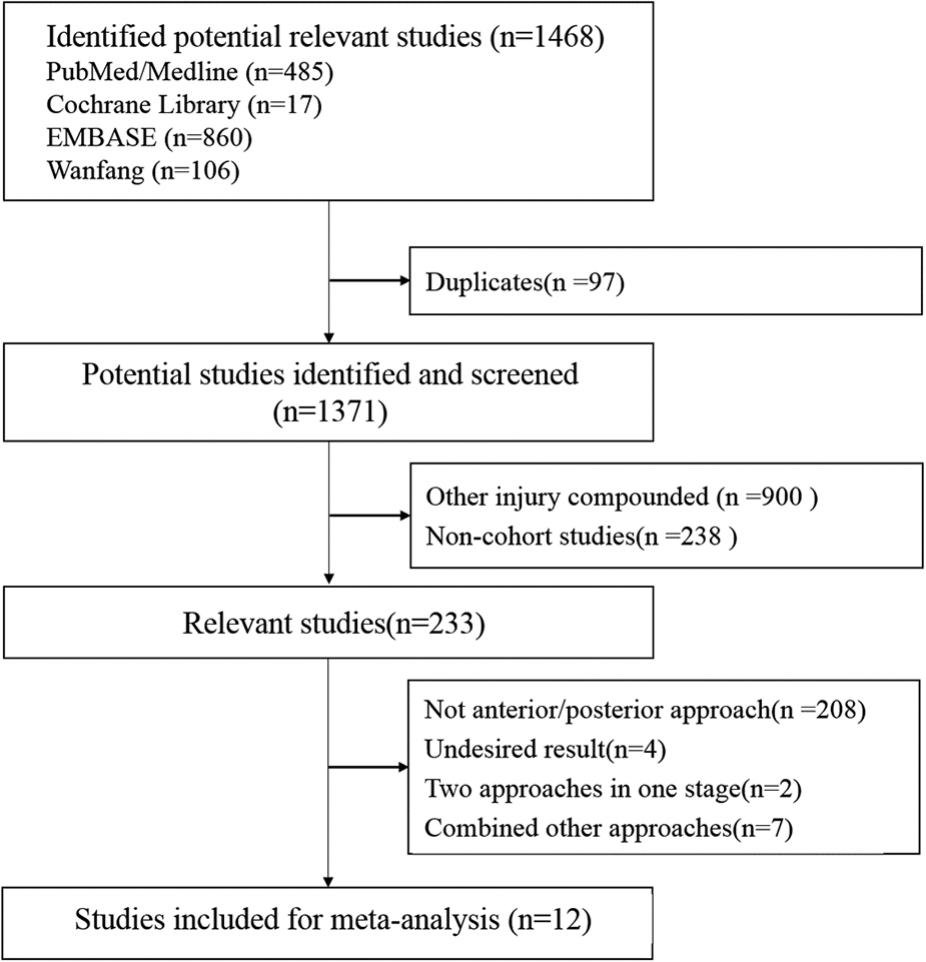
Selection process for meta-analysis of the studies.

A total of 1,468 studies were searched and screened. Finally, 12 articles (10–21) with a total of 452 patients (266 men and 186 women) were included in the study (Figure 1).

The relevant information is given in Table 1. Included articles were all retrospective therapeutic studies, among which seven studies were with a mean age over 60 years. The majority of fractures were type II odontoid fractures (89.6%), and the fresh fractures accounted for 81.4%. The common causes of odontoid fractures were traffic accidents (44.6%) and falling trauma (43.0%), followed by hitting injuries (6.0%) and others. A total of 278 patients underwent surgery through AA; the majority of procedures were performed by a conventional approach (93.2%), while 19 cases were operated via an oral route (12, 18, 20). PA was performed in 174 cases, and most procedures were C1–C2 arthrodesis (95.4%). Among these, pedicle or lateral mass screw fixation (54.8%), cable with bone graft fixation (24.1%) (14, 16, 20), articular screw fixation (16.9%), and splint with bone graft (1.8%) (14, 15) were orderly performed, while one article (21) did not involved the fusion rate but provided other results.

**Table 1:** Characteristics of the 12 included studies.

Study and publication year	Mean age (y)	Gender		Fracture type		FU (m)	Anterior approach		Posterior approach	
		M	F	II	III		Usual screw + oral way	No. of F	C1–2 + OC fusion	No. of F
Andersson 2000 (10)	78.0	7	11	15	3	24.0	11	9	7	7
Omeis 2009 (11)	79.9	11	18	29	0	18.0	16	15	11 + 2	13
Fujii 1988 (12)	34.0	21	7	22	6	Unknown	11 + 9	16	8	8
Ziai 2000 (13)	57.0	12	8	14	6	3.0	13	5	7	4
Mashhadinezhad 2012 (14)	33.0	33	13	46	0	9.0	15	13	31	28
Pointillart 1994 (15)	54.0	45	23	68	0	6.0	49	47	19	15
Platzer 2007 (16)	71.4	25	31	48	8	1.5	37	33	19	19
Konieczny 2012 (17)	64.5	22	16	32	6	6.0	13	10	25	25
Chiba 1996 (18)	35.0	50	16	52	14	12.0	45 + 9	49	12	12
Scheyerer 2013 (19)	81.7	14	19	33	0	31.1	17	10	16	15
Steltzlen 2013 (20)	60.1	15	7	19	3	6.0	14 + 1	9	4 + 3	6
Ardeshiri 2013 (21)	81.1	11	17	27	1	41.4	18	Unknown	7 + 3	Unknown

M, male; F, female; FU, follow-up time; No. of F, number of fusions; OC, occipitocervical fusion.

**Figure 2:**
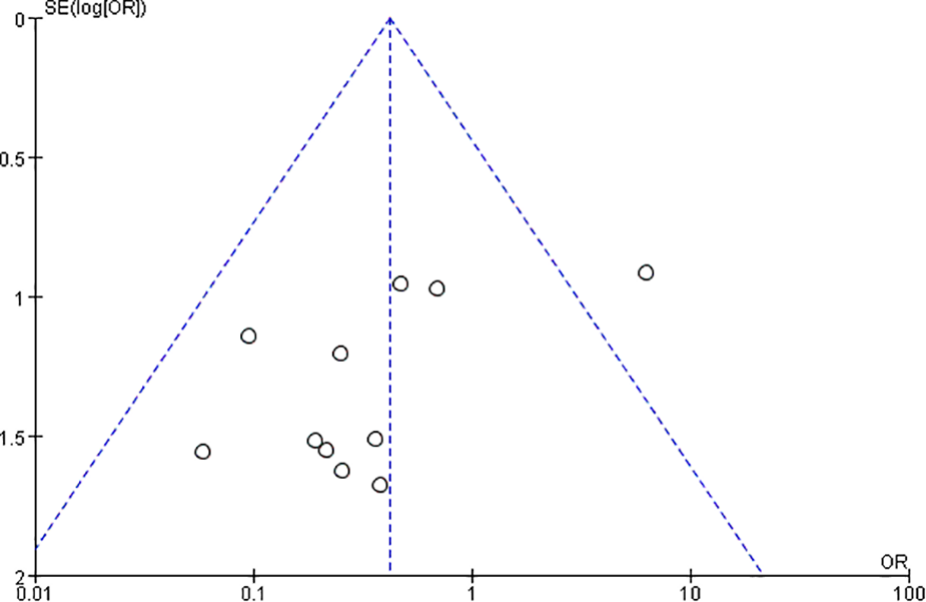
Funnel plot of the postoperative fusion rates of included studies.

The NOS score for each included study is listed in Table 2. All 12 studies had a score of more than 6, of which eight studies were of high quality. A funnel plot was constructed to evaluate publication bias (Figure 2), which, on the whole, suggested a little bias.

**Table 2:** NOS for quality evaluation of included studies.

Studies	Selection of AA and PA				Comparability		Outcome			Total score
	Representativeness of the exposed cohort	Selection of the nonexposed cohort	Ascertainment of exposure	Demonstration that the outcome of interest was not present at the start of the study	Study controls for age	Study controls for any additional factor	Assessment of outcomes	Follow-up long enough	Adequacy of follow-up	
Andersson et al. (10)	0	1	1	1	0	0	1	1	1	6
Omeis et al. (11)	0	1	1	1	0	0	1	1	1	6
Fujii et al. (12)	0	1	1	1	1	1	1	1	1	8
Ziai and Hurlbert (13)	0	1	1	1	1	0	1	1	1	7
Mashhadinezhad et al. (14)	0	1	1	1	1	1	1	1	1	8
Pointillart et al. (15)	0	1	1	1	0	0	1	1	1	6
Platzer et al. (16)	0	1	1	1	0	1	1	1	1	7
Konieczny et al. (17)	0	1	1	1	1	0	1	1	1	7
Chiba et al. (18)	0	1	1	1	1	0	1	1	1	7
Scheyerer et al. (19)	0	1	1	1	0	0	1	1	1	6
Steltzlen et al. (20)	0	1	1	1	0	1	1	1	1	7
Ardeshiri et al. (21)	0	1	1	1	0	1	1	1	1	7

NOS, Newcastle–Ottawa quality assessment scale; AA, anterior approach; PA, posterior approach.

### 3.2. Meta-analysis of the fusion rate

**Figure 3:**
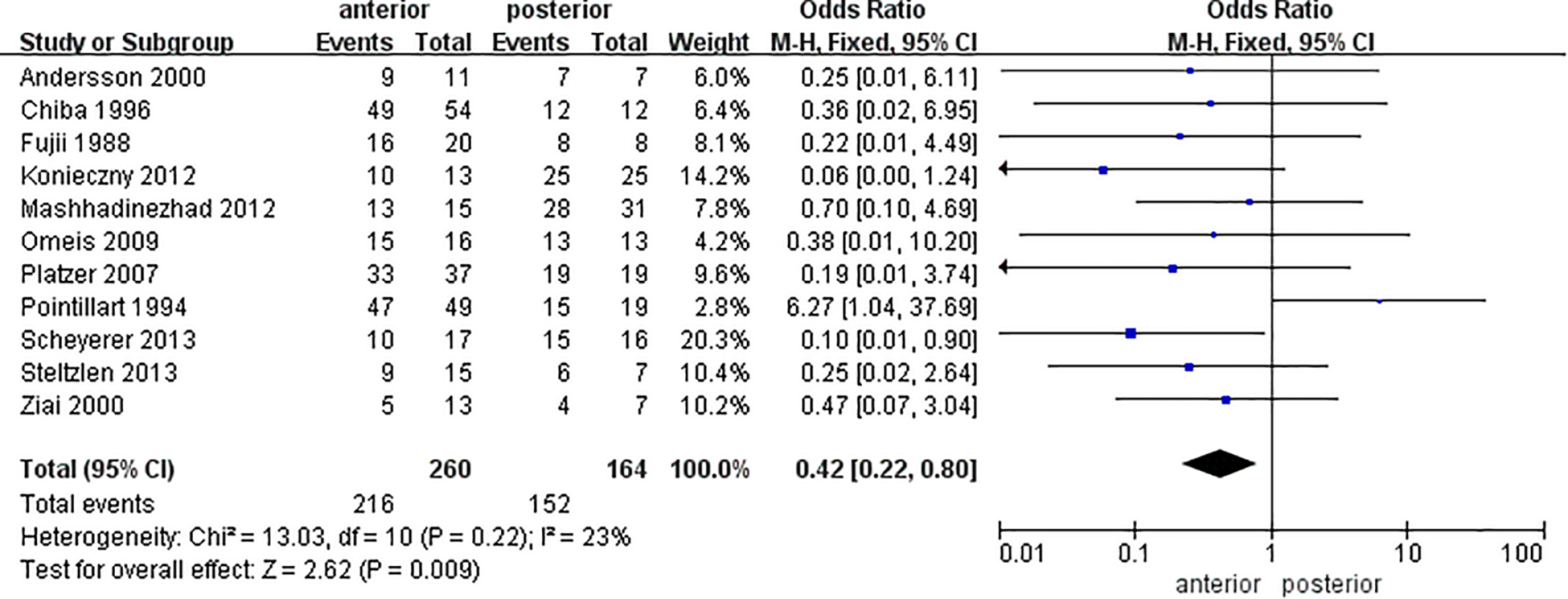
Forest plot of the meta-analysis of fusion rates of AA and PA.

The fusion rates involved in 11 studies were analyzed, where the average fusion rates were 77.5 ± 17.9% in AA and 91.4 ± 13.5% in PA. The meta-analysis for the fusion rate (Figure 3) revealed a statistical difference between the two approaches [OR = 0.42 (0.22, 0.80), *P* = 0.009, *I*^2 ^= 23%].

### 3.3. Subgroup analysis of the fusion rate

**Figure 4:**
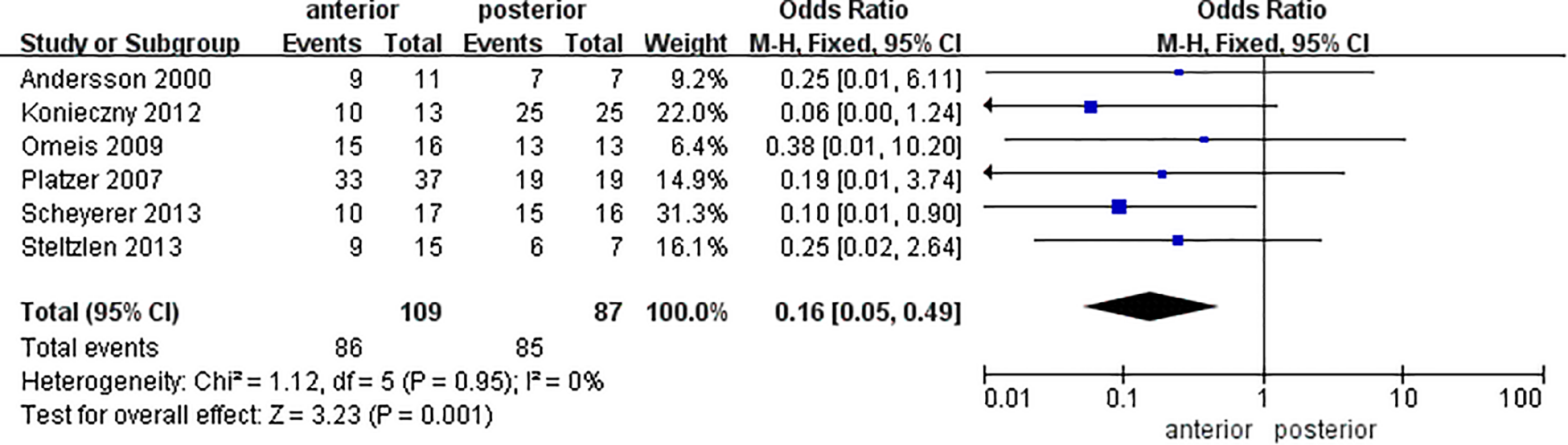
Forest plot of the meta-analysis of postoperative fusion rates in the elderly group.

According to stratification by age, the fusion rate in the elderly group (mean age >60 years) showed a statistical difference between AA and PA [OR = 0.16 (0.05, 0.49), *P* = 0.001, *I*^2^ = 0%], while it was indifferent between the two procedures in the adult group [OR = 0.90 (0.38, 2.13), *P* = 0.81, *I*^2^ = 36%] (Figure 4). A total of 224 patients (7 studies) (10, 11, 16, 17, 19–21) were in the elderly group, consisting of 127 people via AA and 97 cases via PA. Table 1 shows that the proportion of AA ranged from 34.2% to 68.2% in the elderly group and from 32.6% to 81.8% in the adult group. The surgery approach selection between the two age groups showed a statistical difference by a nonparametric test (*Z* = 2.08, *P* = 0.038), and it was considered that elderly might be more likely to choose PA.

### 3.4. Comparison of postoperative mortality

Five articles referred to postoperative mortality (11, 13, 16, 19, 20), and the mortality rates of AA (5.0%) and PA (2.3%) showed no statistical difference (*χ*^2 ^= 1.442, *P* = 0.230).

Omeis et al. (11) reported three cases of death during an 18-month follow-up in 29 patients (16 cases in AA and 13 in PA), consisting of two cases with AA and one with PA, of which one died of acute myocardial infarction and the others died of medically unrelated issues. Ziai and Hurlbert (13) described three cases of death (one case in AA), of which two cases died of spinal cord injury, and one died of a medically unrelated event. Platzer et al. (16) reviewed 56 cases, where three died cases were from the AA group and one was from the PA group, who were all related to the operation, with one died of cardiac arrest, two died of severe respiratory failure, and one died of pulmonary embolism. Scheyerer et al. (19) reported four died cases out of 33 patients (17 cases in AA) immediately died after discharge, all in the AA group. Steltzlen et al. (20) reported four died cases out of 15 patients in the AA group, but the specific causes were unidentified.

### 3.5. Analysis of complications

Nine studies referred to complications, with a rate of 9.7%, where the rates in AA and PA groups were comparable (*χ*^2 ^= 0.918, *P* = 0.338) (Table 3).

**Table 3:** Ttypes and numbers of complications in AA and PA.

Complications	AA	PA	Total
Screw replantation and dislocation	16	3	19
CSF leakage	0	1	1
Spinal cord injury	1	1	2
Superior laryngeal nerve injury	4	0	4
Pharyngalgia	2	0	2
Wound infection	0	3	3
Urinary infection	1	0	1
Liquidizing	1	0	1
Redisplacement	2	0	2
Venous plexus bleeding	0	5	5
Respiratory failure	2	0	2
Cardiac arrest/infarction	1	1	2
Total	30	14	44

AA, anterior approach; PA, posterior approach; CSF, cerebrospinal fluid.

The majority of adverse events were intraoperative screw repositioning or loosening (48.7%), venous plexus bleeding (12.8%), and wound infection (7.7%), where screw displacement (64.0%) was the prevalent complication in the AA group, while venous plexus injury (28.4%), wound infection (21.4%), and screw repositioning (21.4%) were prevalent complications in PA. Five cases of plexus hemorrhage were all reported by Scheyerer et al. (19). Ziai et al. (13) reported two cases of spinal cord injuries in AA and PA groups and one case of urinary tract infection in the AA group.

### 3.6. Quality of life after AA and PA

Here, only one study (14) involved surgery time, which reported a mean operated time of 65 min in the AA group, less than the 118 min in the PA group. One study (21) that referred to the neurological function score was performed by Ardeshiri et al., who assessed it by the ASIA grade. Platzer et al. (16) assessed the postoperative efficacy with the “excellent–good–fair–poor” grade, where 37 cases were with the 27–8–2–0 number in the AA group and 19 cases were with the 5–4–8–2 number in the PA group. As to the operated segmental motion, Platzer et al. (16) reported that 11 cases were facing range-of-motion limitation in the AA group and all cases (19 cases) suffered from it in the PA group; a similar viewpoint was addressed by Scheyerer (19).

## 4. Discussion

The nonunion of an odontoid fracture and the consequent pseudoarthrosis possibly led to compression of the spinal cord and a high incidence of morbidity (22). Bohler et al. advocated that surgical intervention can improve the fusion rate by 26%–80% compared to conservative treatment due to the traumatic instability of type II and type III fractures (23). Some authors believed that PA was a stable strategy with a reliable fusion rate, while Dailey et al. considered that AA could also provide an acceptable fusion rate, with the superiority of less range of motion loss, minimal muscle stripping, and reduction of hospitalization time (24).

In our data, both type II and type III fractures were included, most of which were type II (83.8%). In contrast, few analytical studies on type I fractures have been published because of the absence of significant surgical indications. Radiology was the most intuitive method for postoperative fusion (25), and the evaluation of its outcomes was dependent on its measurements. In this study, by consolidating a large number of articles through meta-analysis, it was confirmed that the fusion rates were higher through PA than AA, which was consistent with previous publications (7, 16).

It was believed age might be the critical factor affecting the outcomes of surgery (9). The study conducted by Nourbakhsh et al. confirmed that the fusion rate did not differ between AA and PA in patients younger than 55 years (26). Biomechanically, PA provides a stable basis through joint fusion and cervical-motion restriction. In contrast, the anterior technique was performed through odontoid screw fixation without bone implantation, causing, to a certain extent, damage to the vessels of the fracture segment. In the older population, osteoporosis and poor blood supplementation further resulted in a lower healing rate (25). Therefore, the difference in fusion rates between AA and PA in the elderly indicated that the latter was a better selection for them.

Ardeshiri et al. (21) reported that the incidence of postoperative complications was 7% in the elderly, with a mortality rate of 0%–57%. Montesano and Osti publicized that the incidence of dysphagia caused by anterior screw fixation was 17%–35% and that of pneumonia was 14%–19% (4, 27), while the incidence of PA-related infection was 33% and that of pneumonia was 17%. In this study, we proved that there was no statistical difference in the number of adverse events between AA and PA but with disparities in the kinds of complications. Platzer et al. believed the incidence of complications was higher in the elderly group (16). The nonparametric test in this study also showed that elderly might suffer from a higher complication rate (*P* < 0.001), both in AA and PA, which was consistent with previous publications, suggesting that elderly should pay more attention to this issue.

Mortality was analyzed in five studies, with rates of 0%–26.7%, which was associated with myocardial infarction, pulmonary embolism, respiratory failure, and spinal cord injury. Therefore, close monitoring of the physiological state and cardiopulmonary function should be emphasized during the perioperative period. The mean age of more than 60 years old was tabulated here in four out of the five articles (11, 16, 19, 20), which predicted that the incidence of postoperative mortality in the elderly was possibly higher than that in adults. However, with the few positive results in this study, the correlation between nonfusion and death was hard to be addressed. Given that there were no death event cases in the other seven studies at the end of follow-up, a statistical difference in the mortality rates between AA and PA could not be considered.

There has always been a controversy between postoperative complications and nonunion. Schatzker et al. suggested that nonfusion would lead to a series of adverse events such as spinal cord injury (28), whereas others reported that no obvious symptoms were found, although with as high as 33% nonfusion rate postoperatively. The follow-up study (mean of 5.6 years) on five older patients conducted by Hart et al. showed there was no case trapped in spinal cord lesions with adverse nonfusion, although with the suspicion of paralysis (29). This article believed that age was related to the rate of fusion and complications, while it failed to draw a correlation between nonfusion and complications because of the low incidence of complications, which may be consistent with the previous reports.

Despite limited literature referring to the operative time and segmental range of motion, it was thought that the anterior technique involved less invasion and short operation time, together with more preservation of segmental motion. Commonly, the consumable material of AA was simpler than PA on internal fixation, which reflected that AA might be an inexpensive alternative with potentially shorter hospitalization time. Consequently, this surgical approach is likely to reduce the overall charge (24), while a further cost–benefit analysis must be performed individually.

Some limitations should be noted in this study. The specific types of fractures may affect the rate of fusion and complications (6), but the included studies failed to extract the information on the proportion of type II or type III fractures, which led to a heterogeneity in subgroup analysis. Then, it was the mean age instead of the individual data that differed between the elderly and adult groups; as a result, the probability of abnormal distribution or crossover between the two age groups would bring reporting bias. The inconsistency and large span of follow-up among enrolled studies would also affect the evaluation of the postoperative fusion rate. Finally, the reports on outcomes of odontoid fractures in low-level quality would generate inevitable bias.

## 5. Conclusion

By performing the meta-analysis based on 12 studies, we found that PA acquired a higher fusion rate of odontoid fractures, while AA may be superior in the operation time and segmental motion retention. The older population preferred to select PA, although the fusion rate was of no statistical difference between adults and the elderly. Most adverse events were screw repositioning or loosening in the AA group, while venous plexus injuries and wound infections were more common in the PA group. There was no statistical difference in the incidence of complications and the rate of mortality between the two approaches. The correlation between nonfusion and complications remained unidentified. In sum, when the fusion rate was focused on first, the posterior approach would be preferred and more reliable.

## Data Availability

The original contributions presented in the study are included in the article/Supplementary Material, further inquiries can be directed to the corresponding author.

## Supplementary material

SUPPLEMENTARY TABLE S1

Supplemental Digital Content, which was relevant data about this study.
